# Terrestrial Origin of Viviparity in Mesozoic Marine Reptiles Indicated by Early Triassic Embryonic Fossils

**DOI:** 10.1371/journal.pone.0088640

**Published:** 2014-02-12

**Authors:** Ryosuke Motani, Da-yong Jiang, Andrea Tintori, Olivier Rieppel, Guan-bao Chen

**Affiliations:** 1 Department of Earth and Planetary Sciences, University of California, Davis, Davis, California, United States of America; 2 Laboratory of Orogenic Belt and Crustal Evolution, Ministry of Education, Department of Geology and Geological Museum, Peking University, Beijing, People’s Republic of China; 3 State Key Laboratory of Palaeobiology and Stratigraphy (Nanjing Institute of Geology and Palaeontology, Chinese Academy of Sciences), Nanjing, Jiangsu Province, People’s Republic of China; 4 Dipartimento di Scienze della Terra, Università degli Studi di Milano, Via Mangiagalli, Milano, Italy; 5 Center of Integrative Research, The Field Museum, Chicago, Illinois, United States of America; 6 Department of Research, Anhui Geological Museum, Hefei, Anhui Province, People’s Republic of China; University of Pennsylvania, United States of America

## Abstract

Viviparity in Mesozoic marine reptiles has traditionally been considered an aquatic adaptation. We report a new fossil specimen that strongly contradicts this traditional interpretation. The new specimen contains the oldest fossil embryos of Mesozoic marine reptile that are about 10 million years older than previous such records. The fossil belongs to *Chaohusaurus* (Reptilia, Ichthyopterygia), which is the oldest of Mesozoic marine reptiles (ca. 248 million years ago, Early Triassic). This exceptional specimen captures an articulated embryo in birth position, with its skull just emerged from the maternal pelvis. Its headfirst birth posture, which is unlikely to be a breech condition, strongly indicates a terrestrial origin of viviparity, in contrast to the traditional view. The tail-first birth posture in derived ichthyopterygians, convergent with the conditions in whales and sea cows, therefore is a secondary feature. The unequivocally marine origin of viviparity is so far not known among amniotes, a subset of vertebrate animals comprising mammals and reptiles, including birds. Therefore, obligate marine amniotes appear to have evolved almost exclusively from viviparous land ancestors. Viviparous land reptiles most likely appeared much earlier than currently thought, at least as early as the recovery phase from the end-Permian mass extinction.

## Introduction

Viviparity allows maternal maintenance of the embryonic environment, and is known across bony fishes, elasmobranchs, amphibians, reptiles, and mammals [1]. It independently evolved at least 141 times in the vertebrates, of which 108 are found in squamate reptiles [2]. The oldest fossil record of viviparity in vertebrates belongs to a placoderm 'fish' from the Devonian, approximately 380 million years old [3], but that for amniotes is younger at about 280 million years old [4], in a Permian marine reptile *Mesosaurus* that lived in an inland sea [5]. Viviparity is considered mostly a terrestrial feature in amniotes [6]. However, viviparity is also a necessary feature in obligatory marine amniotes, such as some Mesozoic marine reptiles [7]–[13], which could not walk on land or lay eggs in the sea [8], [9]. This raises the question of whether viviparity in obligatory marine Mesozoic reptiles was inherited from their respective terrestrial ancestors, or evolved after each lineage invaded the sea as an aquatic adaptation, as has traditionally been thought.

Ichthyopterygia is a group of Mesozoic marine reptiles known for a fish-shaped body profile [14] and enormous eyes [15] in derived forms. They are also known for being viviparous: at least six ichthyopterygian genera, spanning the Middle Triassic to Early Cretaceous, have fossil records of embryos [11]–[13], [16], [17]. However, the reproductive biology of the earliest ichthyopterygians, which lived in the late Early Triassic (ca. 251–247 million years ago), was not known.

**Figure 1:**
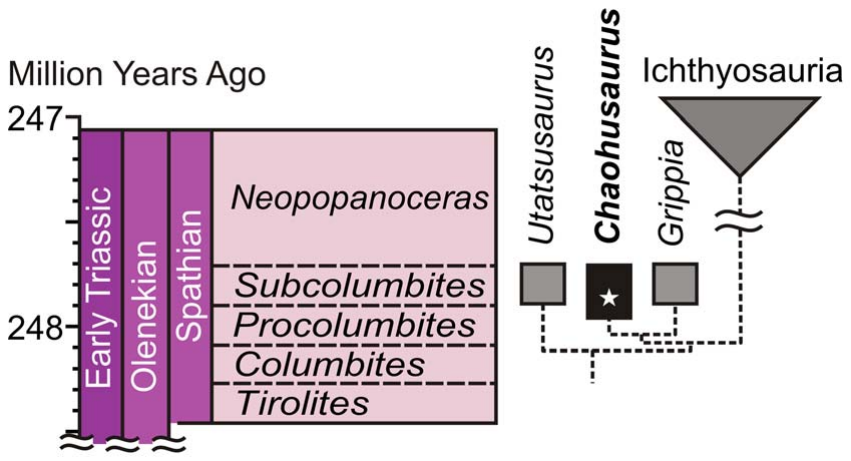
Stratigraphy and phylogeny of the earliest ichthyopterygians, with a reconstruction of the typical birth posture in derived members. *Chaohusaurus* has the oldest stratigraphic record of the three Early Triassic genera. Star indicates the stratigraphic position of the present specimen. Time scale was drawn using TS Creator 6.1.2 that follows [4]. See fig. S1 for a high resolution image.

**Figure 2:**
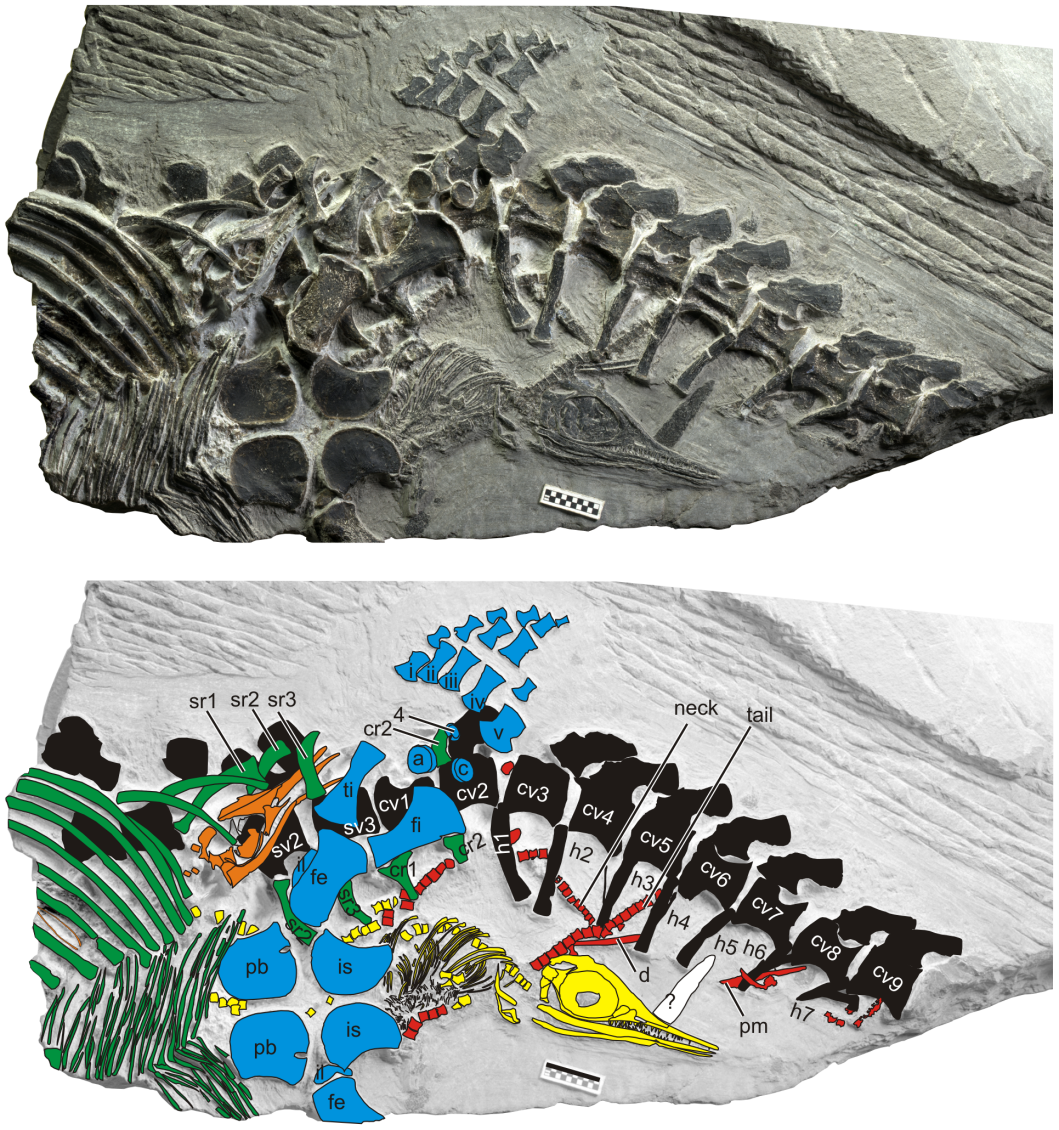
The maternal specimen with three embryos. Color coding indicates: black, maternal vertebral column, including neural and haemal spines; blue, maternal pelvis and hind flipper; green, maternal ribs and gastralia. Embryos 1 and 2 are in orange and yellow, respectively, whereas neonate 1 is in red. Scale bar is 1 cm. Abbreviations: i-v, metatarsals; 4, fourth distal tarsal; a, astragalus; c, calcaneum; cr, caudal rib; cv, caudal vertebra; d, dentary; fe, femur; fi, fibula; h, haemal spine; il, ilium; is, ischium; pb, pubis; pm, premaxilla; sr, sacral rib; sv, sacral vertebra; and ti, tibia. See fig. S2 for a high resolution image.

*Chaohusaurus* is one of the three basal ichthyopterygian genera that unequivocally lived during the Early Triassic [18]. Our recent field excavation [19] yielded more than 80 new skeletons, greatly advancing our knowledge of the earliest ichthyopterygians—there were less than 20 skeletons known of the three genera before. *Chaohusaurus* is not the most basal of the Early Triassic ichthyopterygians [18] but our field survey revealed that the genus had the oldest stratigraphic record of the three (figure 1), extending back at least to the early-middle Spathian (*Procolumbites* Zone), some 248 million years ago [4]. Among the 80 specimens was a partial skeleton that contained embryos, AGM I-1 (Anhui Geological Museum, Hefei, China), which uncovers for the first time the reproductive strategy of the earliest marine reptiles of the Mesozoic (figure 2).

## Materials and Methods

### Specimens

The specimens used in this study (AGM I-1, AGM CHS-5, and AGM CH-628-22) were all collected from a fossil quarry in south Majiashan, Chaohu, Anhui, China, through a joint excavation by AGM, Peking University, University of California, Davis, Università degli studi di Milano, and the Field Museum, with permits from the Ministry of Land and Resources of the People’s Republic of China. The Spathian Nanlinghu formation is exposed in the quarry. All specimens are accessioned at the Anhui Geological Museum in Hefei City, Anhui Province, China. All three specimens are from near the bottom of the *Subcolumbites* zone of the Middle Spathian (Lower Triassic).

The specimen with embryos (AGM I-1) was initially collected while still concealed in the rock as a 'by-catch' of a specimen of the predatory fish *Saurichthys* that was exposed on the same slab (AGM I-2). It was later uncovered in the laboratory by our preparator, so there is no possibility of forgery. It is unlikely that the *Saurichthys* on the same slab was hunting for a newborn *Chaohusaurus*. It did not occupy the same time horizon with the *Chaohusaurus* individuals because there are a few laminae of mudstones between the two.

### Taxonomy

The three new specimens are assigned to *Chaohusaurus* for the following reasons. Most importantly, *Chaohusaurus* is the only ichthyopterygian to exhibit delayed ossification of carpals and tarsals relative to metacarpals/metatarsals and phalanges, and this feature is clearly present in all of the specimens used in this study. The three specimens further exhibit the typical dental morphology, vertebral count, and the unique hypophalangeal flippers of *Chaohusaurus*. The current taxonomy holds that *Chaohusaurus geishanensis* is the only species within the genus [18], [20], although specific taxonomy may require a revision as more specimens are prepared.

### Maternal Size

**Figure 3:**
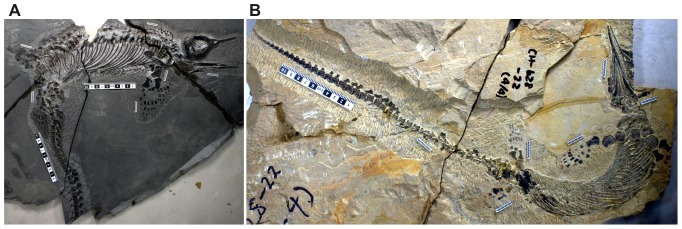
Completeness of the two skeletons used in maternal body size estimation. (A), AGM CHS-5, a nearly complete skeleton that is almost as large as AGM I-1. (B), AGM CH-628-22, a complete skeleton that preserves the tail tip. Large scale bars are 10 cm, and short bars 2 cm.

AGM CHS-5 is a nearly complete skeleton only lacking the tail tip (figure 3A). This specimen is about 1% smaller than AGM I-1 in the length of the second caudal vertebra (10.97 versus 11.16 mm), which we use as the standard for comparing the sizes of incomplete specimens because it has a high exposure rate across the specimens. Its skull is 117.03 mm long, and its skeleton is estimated to be about 100 cm when compensating for the missing tail tip based on the vertebral count and size in CH-628-22, as explained below. We estimated the body and skull lengths of AGM I-1 based on these numbers but they are approximate because no two individuals have strictly identical bone proportions.

### Skeletal Reconstructions

**Figure 4:**
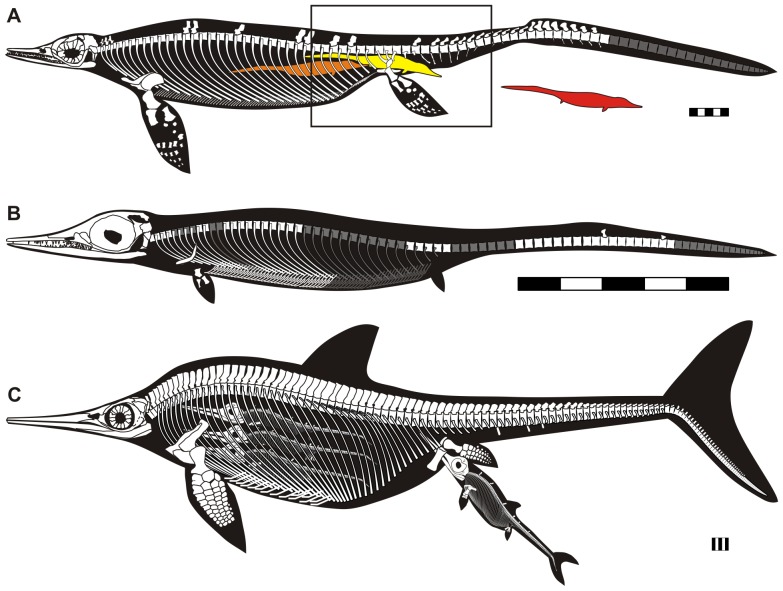
Stylized reconstruction of adult and embryo of *Chaohusaurus* in comparison to a derived ichthyopterygian. (A), adult based on AGM I-1 and CHS-5. Rectangle indicates the approximate range preserved in AGM I-1. Colored silhouettes of embryo are placed in approximate positions of embryos 1 and 2, with embryo 3 displaced to avoid overlap with embryo 2. The extent of the maternal tail tip, in gray, is based on AGM-CH-628-22. Scleral ring is based on AGM-CHS-3. (B), embryo based on embryo 2 and neonate 1 of AGM I-1. Elements in gray are missing. (C), the derived ichthyopterygian *Stenopterygius* with one embryo in birth position and three in body cavity, reconstructed based on SMNS 6293 (Staatliches Museum für Naturkunde, Stüttgart, Germany). Scale bars are 5 cm. See fig. S3 for a high resolution image.

Each panel of figure 4 was made using the following procedures. First, vertebral column was drawn based on the actual measurements of vertebral size and approximate angle at each vertebral position using a script written for R 3.0.2. Missing measurements were interpolated using a local polynomial curve fit to the available data. The missing tail tip of AGM CHS-5 (figure 3A) was estimated by adding the tail tip vertebra based on AGM CH-628-22 (figure 3B), a slightly smaller specimen with a complete tail, before this polynomial fitting. The tip vertebra was linearly scaled up to match AGM CHS-5, and placed at the correct vertebral position. Other elements were traced from a photograph, scaled, rotated, and transposed to fit the vertebral column in a vector drawing software (CorelDraw). Some ribs were interpolated using the linear morphing function of CorelDraw. Body outlines in black are approximate.

## Description

The maternal specimen is incomplete, lacking the skull, anterior trunk, and posterior tail (figure2) because of the way it was collected (see Methods). However, the bones are excellently preserved and well-articulated. Despite the incompleteness, the maternal body size can be estimated from another specimen with almost identical vertebral and pelvic dimensions (AGM CHS-5, figure 3). We estimate the maternal body length to be approximately 100 cm, and the skull length about 12 cm (see Methods).

There are at least three embryos/neonates associated with the maternal skeleton (figure 2), one inside the maternal body cavity (embryo 1), another exiting the pelvic girdle with half of the body still in the maternal body cavity (embryo 2), and the third outside of the maternal body, largely underlying it (neonate 1). The preservation of embryos is exquisite despite the great geologic age. Two of the skulls are articulated, unlike in most fossil embryos [7], [9], [11]. Also, apart from one Jurassic ichthyopterygian specimen (figure 4C), this is the only fossil that captures an articulated embryo in birth position. Embryos 2 and neonate 1 may appear mixed (figure 2) but can be readily delineated from each other because neonate 1 underlies the maternal body whereas embryo 2 is above the right maternal sacral ribs. Also, if the detached tail segment currently assigned to neonate 1 belonged to embryo 2, then the embryo would already be outside the maternal body and there would be no reason for it to be near the maternal pelvic girdle as preserved. Similarly, embryos 1 and 2 occur on different layers, so it is unlikely that the two individuals are confused in figure 2. In addition, based on vertebral size and shape, it is possible to judge that the vertebral columns of the embryos and neonate have caudad orientations except the detached tail segment outside of the maternal body. We estimate the length of an embryo to be about 18 cm, assuming the adult vertebral count (figure 4). The relative embryo to adult size is therefore about 0.18, which is small for an ichthyopterygian but similar to what is known in terrestrial saurians [8], [13].

The skull of embryo 2 is 35 mm long. There are 23 upper and 16 lower teeth preserved in the jaws of embryo 2. When accounting for empty tooth positions, the dental count for the upper jaw is estimated to be about 40 to 45. This is about 10 positions less than the adult condition. All teeth are pointed, although some broken teeth may misleadingly exhibit rounded shape. Adult *Chaohusaurus* are known for heterodonty, with pointed anterior and rounded posterior teeth [18], [20].

## Discussion

A suite of features supports the inference that two of the small individuals are embryos. First, embryo 1 is completely enclosed inside the maternal body cavity and embryo 2 partially enclosed in the maternal pelvic girdle, eliminating a possibility of preservational superimposition. Second, there is no indication of predation or digestion. The bones are not etched by stomach acid and the skeletons are sufficiently well-articulated despite their terminal position. Third, the large relative skull size and small relative flipper length compared to adult ratios [20] indicate immaturity (figure 4). Fourth, ossification of flipper bones is the least extensive among *Chaohusaurus* specimens [20], with the entire autopod unossified. The flipper bones appear angular and stout compared to those of the smallest juvenile known [20], again indicating immaturity. Fifth, tooth shape also suggests immaturity of these individuals. It is known among some extant heterodont lizards [21] and *Chaohusaurus* [20] that the degree of heterodonty is age-related, with younger individuals having progressively isodont (i.e., uniform tooth shape) dentition. Therefore, isodonty seen in this specimen is expected in embryos of *Chaohusaurus*. Finally, the skull suture pattern of embryo 2 is very similar to that of adults, suggesting that it is conspecific with the adults.

Viviparity in extant reptiles is known only among squamates. Despite the traditional four-step evolutionary model from lecithotrophy to placentotrophy, squamate reproductive strategies are almost bimodally divided between oviparity (egg laying), including cases of egg retention up to limb-bud stage, and viviparity involving functional placentation [2], with few intermediate forms [22]. Therefore, viviparity seems to evolve simultaneously with functional placentation in squamates [23]. Given these observations, it would be reasonable if viviparity in *Chaohusaurus* involved a degree of placentation. However, this inference cannot be tested directly with fossil evidence because the soft tissue is not preserved.

Embryo 2 is in birth position but this location alone does not necessitate a death during parturition. A similar case for the Jurassic ichthyopterygian *Stenopterygius* (figure 4C) has been interpreted as postmortem expunging of an embryo, clogging the birth canal, by abdominal gas from decomposition—note that cranially-located embryos were not pushed backed by the gas in the specimen of *Stenopterygius* [13]. However, in the present specimen, neonate 1 lies outside the maternal body in the present specimen, suggesting that the mother had already given birth to at least one offspring before it died. Placement of embryos 1 and 2 near the pelvic girdle, respectively, suggests that embryos were at least full term. Considering these factors, we conclude that the mother likely died in labor. Given that the rock containing the fossil is marine, parturition most likely occurred underwater.

Both articulated skulls are pointing caudally, and so is the disarticulated skull of neonate 1 (figure 2). We hereafter define directions based on the mother, e.g., caudad means toward the maternal tail tip. It is likely that newborns were expelled headfirst (figure 4A), given the uniform orientation of the embryonic skulls and the lack of room for reorientation of embryos. It is unlikely that all three individuals represent a breech condition. For example, if a possibility of breech is, say, 10%, then chances of having three consecutive breech births is only 0.1%. A similar condition in the basal whale *Maiacetus* was interpreted as evidence for terrestrial birth [24], although anatomical differences between the two forms prevent a direct comparison. It is generally thought that embryos of aquatic amniotes, including whales, sea cows, and ichthyopterygians, are born tail-first (figure 4C), possibly to avoid suffocation during parturition [9], [25], [26]. However, at least some newborns are expelled headfirst in Yellow-bellied Sea Snake (*Pelamis platura*) [27], White Whale (*Delphinapterus leucas*) [28], and the derived ichthyosaurian *Stenopterygius* [13], although the majority of individuals are born tail-first. These cases may be exceptions but nevertheless establish that headfirst birth in water is possible even in air-breathers. Therefore, the caudad skull orientation of embryonic *Chaohusaurus* does not necessarily suggest birth on land, especially if this mother died in labor underwater as preservational evidence suggests.

Although a case for terrestrial birth cannot be established in *Chaohusaurus*, the uniformly caudad skull orientation of its embryos does suggest that viviparity in Ichthyopterygia most likely evolved in their ancestor on land, where caudad embryonic skull orientation during parturition is the norm. The small relative size of embryos, comparable to the mean terrestrial proportions as pointed out earlier, supports this inference. Thus, the craniad orientation of the embryonic skull (figure 4C) is a derived condition within Ichthyopterygia, probably known only in its subclade Ichthyosauria [18]. The fossil record shows that ichthyosaurs as basal as *Mixosaurus* of the Middle Triassic [11] already had cranially-oriented embryonic skulls. We interpret this secondary change in skull orientation as an aquatic adaptation, whereas viviparity itself is an inherited terrestrial feature that happened to help the clade become obligatorily marine. Therefore, viviparity is an exaptation in ichthyopterygians [29].

The caudad embryonic skull orientation during underwater parturition may have led to high mortality in early marine invaders [7]. If so, the current fossil has a rare preservation of an embryo in birth posture, together with a deceased neonate because of such high mortality. However, this inference remains speculative until additional evidence is found. A similar case was reported for the freshwater reptile *Hyphalosaurus* [30].

There is no evidence for a marine origin of viviparity in Mesozoic marine reptiles despite the traditional view. Two clades other than Ichthyopterygia have fossil records of viviparity, viz., Sauropterygia [7], [8] and Mosasauroidea [9]. The embryos of the sauropterygian *Keichousaurus* are preserved with their skulls pointing caudally without a clear sign of vertebral curling [7], as in *Chaohusaurus*. This condition strongly indicates a terrestrial origin of viviparity in Sauropterygia. It was suggested that birth in *Keichousaurus* was expedited by its flexible pelvic girdle, resulting from aquatic adaptation [7]. Such an aquatic adaptation may speed up the birth process as suggested but evidence from extant reptiles is currently lacking to support this hypothesis. Also, this factor may not be relevant to those species with many small embryos in a liter because each newborn is small compared to those of a species with a liter size of one for a given maternal size. At least, the flexible girdle is clearly not a mandatory structure for viviparity because most viviparous reptiles are terrestrial [2]. The presence of curled-up embryos in other Triassic sauropterygians, such as *Neusticosaurus* [31] and *Lariosarus* [10], suggests that the reproductive strategy of these amphibious [8] marine reptiles may have been variable. Such a variability within a clade is possible given that at least three species of extant lizards have both viviparous and oviparous populations [22]. Embryos of the mosasauroid *Carsosaurus* are preserved curled-up, with their heads inclined cranially [9]. Their tails are positioned more cranially than their respective skulls, making tail-first birth unlikely. They may have been born curled-up, as in some extant lizards that give birth on land. Mosasauroids are squamates [32], which are known for unusually high abundance of viviparity [2]. The fossil record of viviparity in this group is at least 30 million years older than that for *Carsosaurus* [33]. Therefore, viviparity in mosasauroids probably was inherited from their land ancestor.

*Hyphalosaurus* from the Cretaceous of China is another example of viviparous aquatic reptile, although it lived in freshwater [30]. A case is known where two terminal embryos within the maternal body cavity were straightened while the others still remained curled, most likely in their egg sacs [30]. Therefore, its viviparity is similar to that of some living squamates, where birth posture varies [34]. Its egg membrane microstructure is also similar to that of squamates [35]. It is unclear how this example relates to the present case of *Chaohusaurus*, for which there is no evidence of embryonic body curling at this point—no ichthyopterygian embryo has been found with a curled body posture.

The marine origin of viviparity is also unknown among extant obligate marine amniotes. Viviparity likely evolved only once in mammals [36], so all marine mammals inherited it from their land ancestors. The only extant obligatory marine reptiles are hydrophiine sea snakes. Note that we consider only those animals that feed almost exclusively in the sea as marine animals [37]. The origin of hydrophiine viviparity had been ambiguous [38]–[40], but a recent molecular phylogenetic study clarified that they are a part of a viviparous clade whose basal members are all terrestrial [41]. Therefore, it is most likely that their viviparity also evolved on land.

Overall, no case is known for the marine origin of viviparity in strictly obligatory marine amniotes through time, whether extinct or extant, based on either phylogenetic bracketing or birth postures in basal forms. A possible reason for this absence is temperature. Studies of extant reptiles suggested that viviparity evolved in cold climates, where thermoregulation through maternal behavior leads to high incubation temperature [1], [42], [43]. Behavioral thermoregulation is expected to be less effective in the sea, where temperature is more stable and has higher minimum values than on land [44]. Thus, amniote viviparity is expected to have higher selective advantage on land than in the sea. Fish viviparity is irrelevant to the present discussion because amniote eggs develop on land [9].

Terrestrial origins of viviparity in Mesozoic marine reptiles may be a departure from the conventional wisdom. However, it agrees well with the general knowledge of viviparity in extant amniotes, as discussed above. The null assumption for viviparity in obligate marine amniotes should be that it evolved in terrestrial ancestors and not in the sea, unless evidence to the contrary is found. The oldest fossil evidence of viviparity in land reptiles is at most 125 million years old [33]. However, this seemingly young age is probably because of preservation bias against small terrestrial vertebrates [45]. Both Ichthyopterygia and Sauropterygia most likely evolved from viviparous land ancestors in the Early Triassic, at least as early as 248 million years ago. Therefore, viviparity may have already been common among terrestrial reptiles during the recovery phase from the end-Permian mass extinction.

## Supporting Information

Figure S1 High resolution version of Fig. 1.
(PDF)

Figure S2 High Resolution Version of Fig. 2.
(PDF)

Figure S3 High resolution version of Fig. 4.
(PDF)
