# Supplementary figures and images for: Terrestrial Origin of Viviparity in Mesozoic Marine Reptiles Indicated by Early Triassic Embryonic Fossils
Source: PLoS One. 2014 Feb 12;9(2):e88640. doi: 10.1371/journal.pone.0088640 (PMC3922983; doi:10.1371/journal.pone.0088640)

Figure S1. High resolution version of Fig. 1.

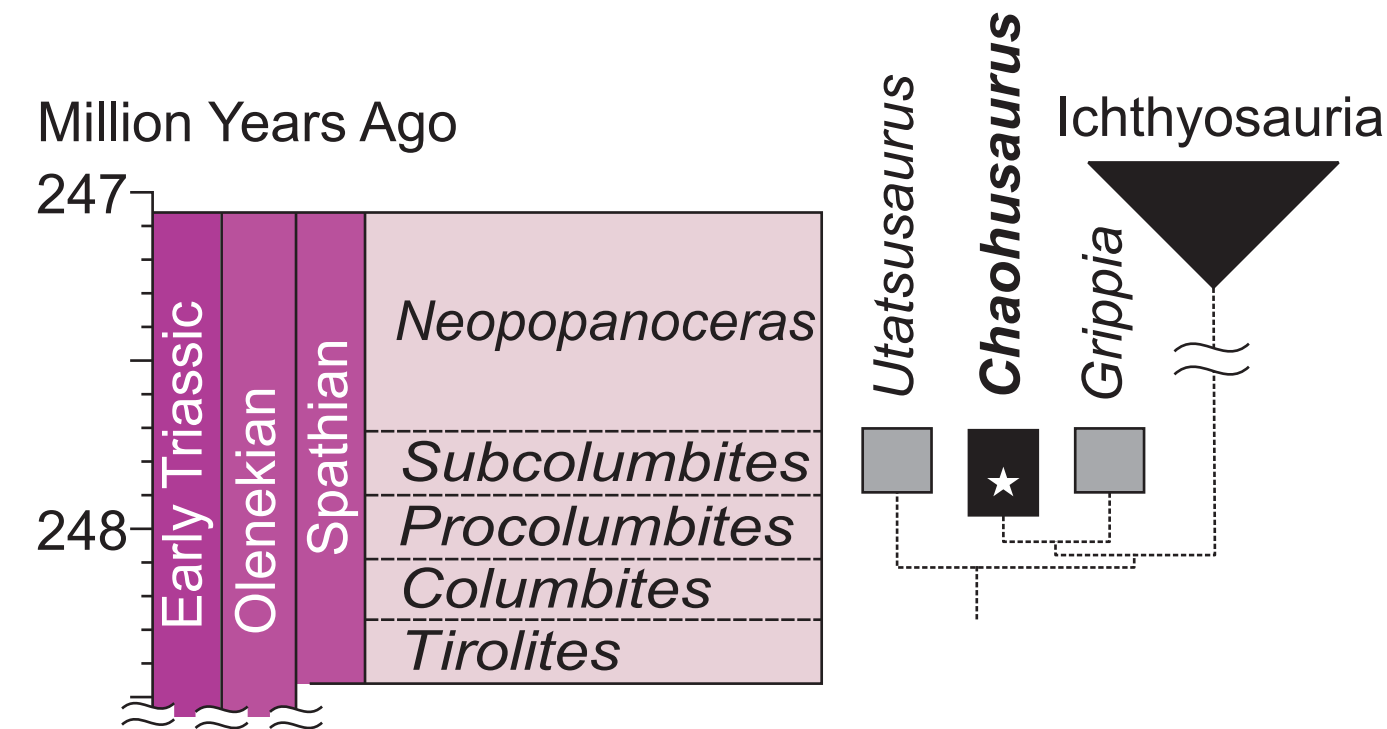

Supplement: Figure S1 — High resolution version of Fig. 1. (PDF) [file pone.0088640.s001.pdf]

Figure S2. High resolution version of Fig. 2.

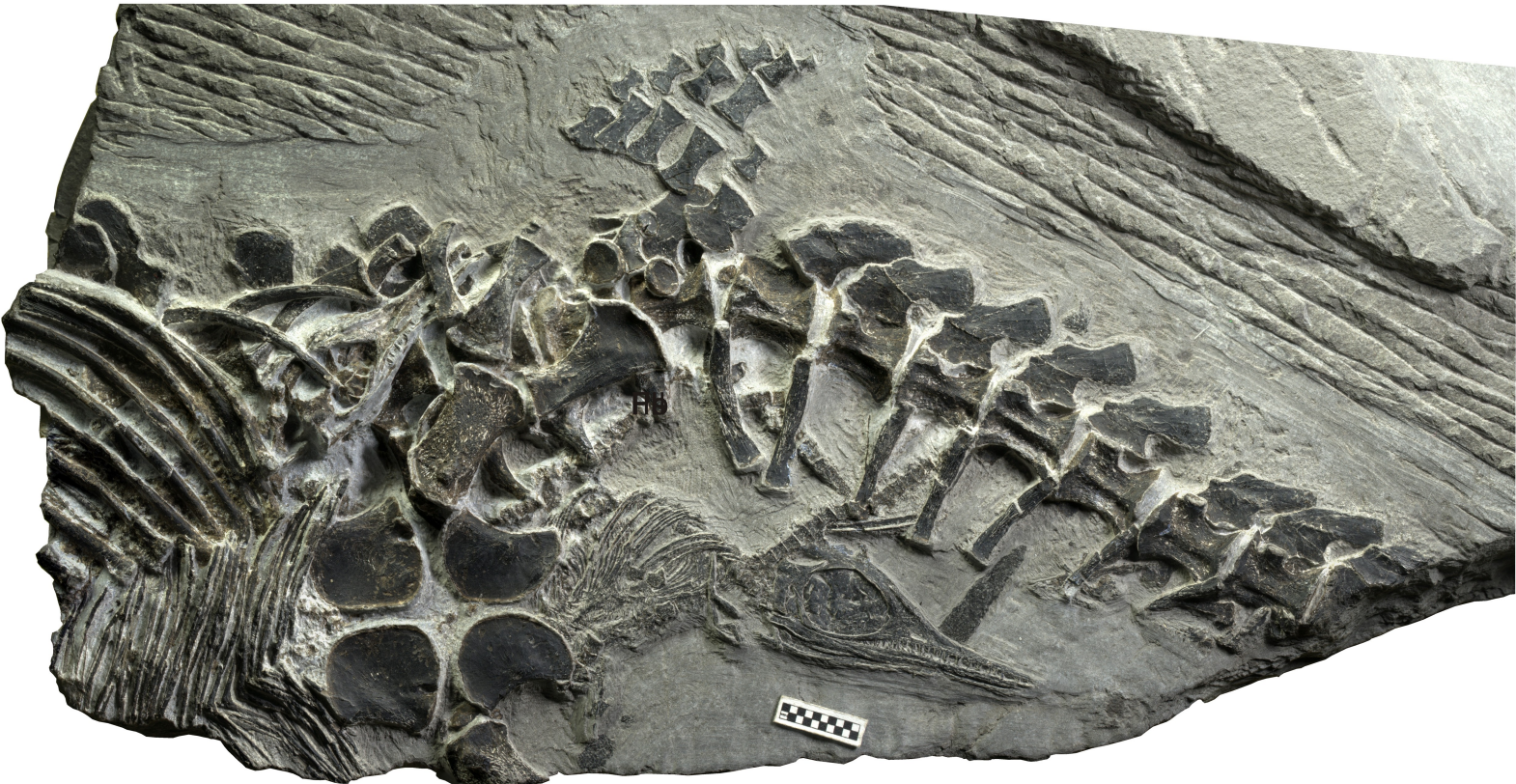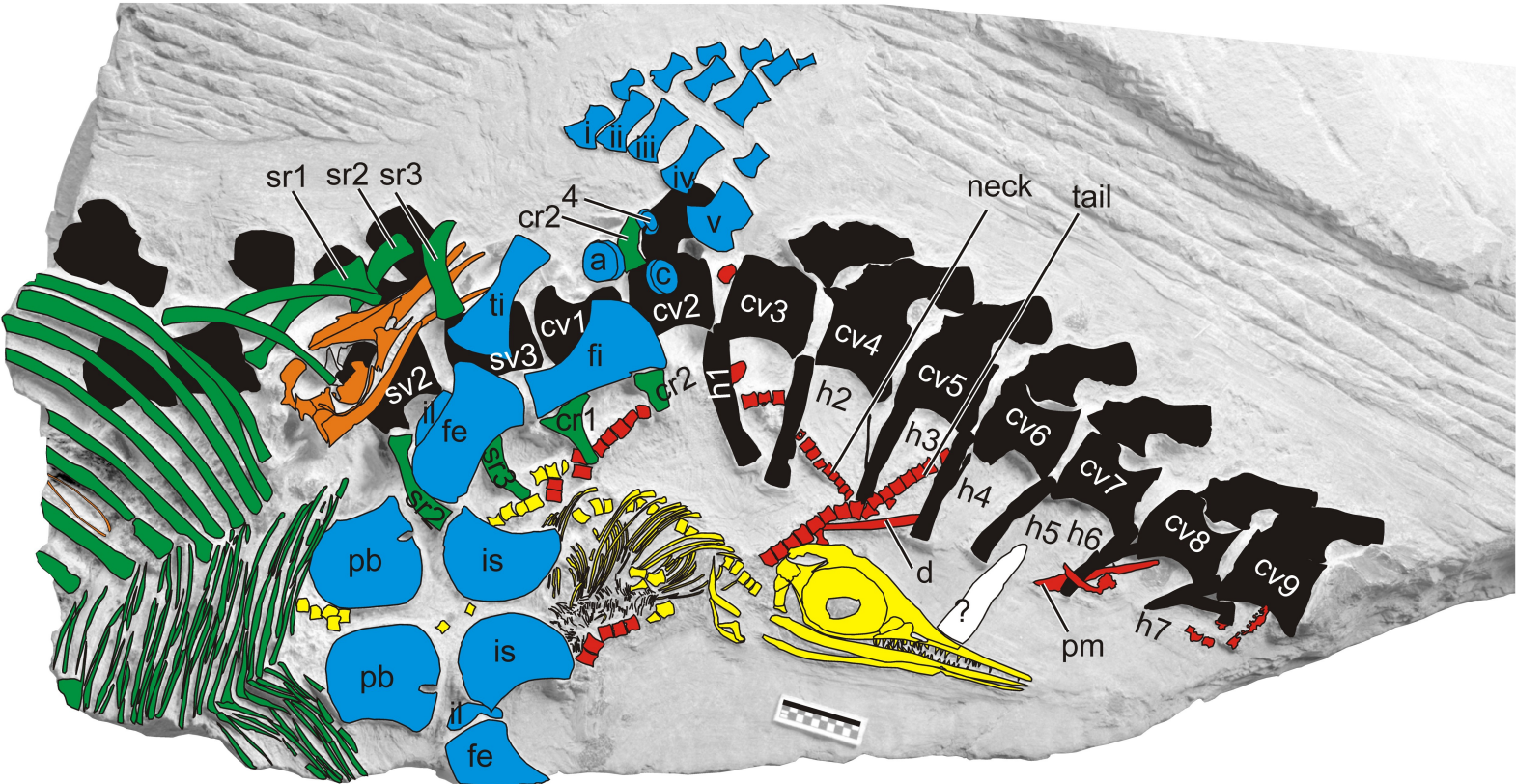

Supplement: Figure S2 — High Resolution Version of Fig. 2. (PDF) [file pone.0088640.s002.pdf]

Figure S3. High resolution version of Fig. 3.

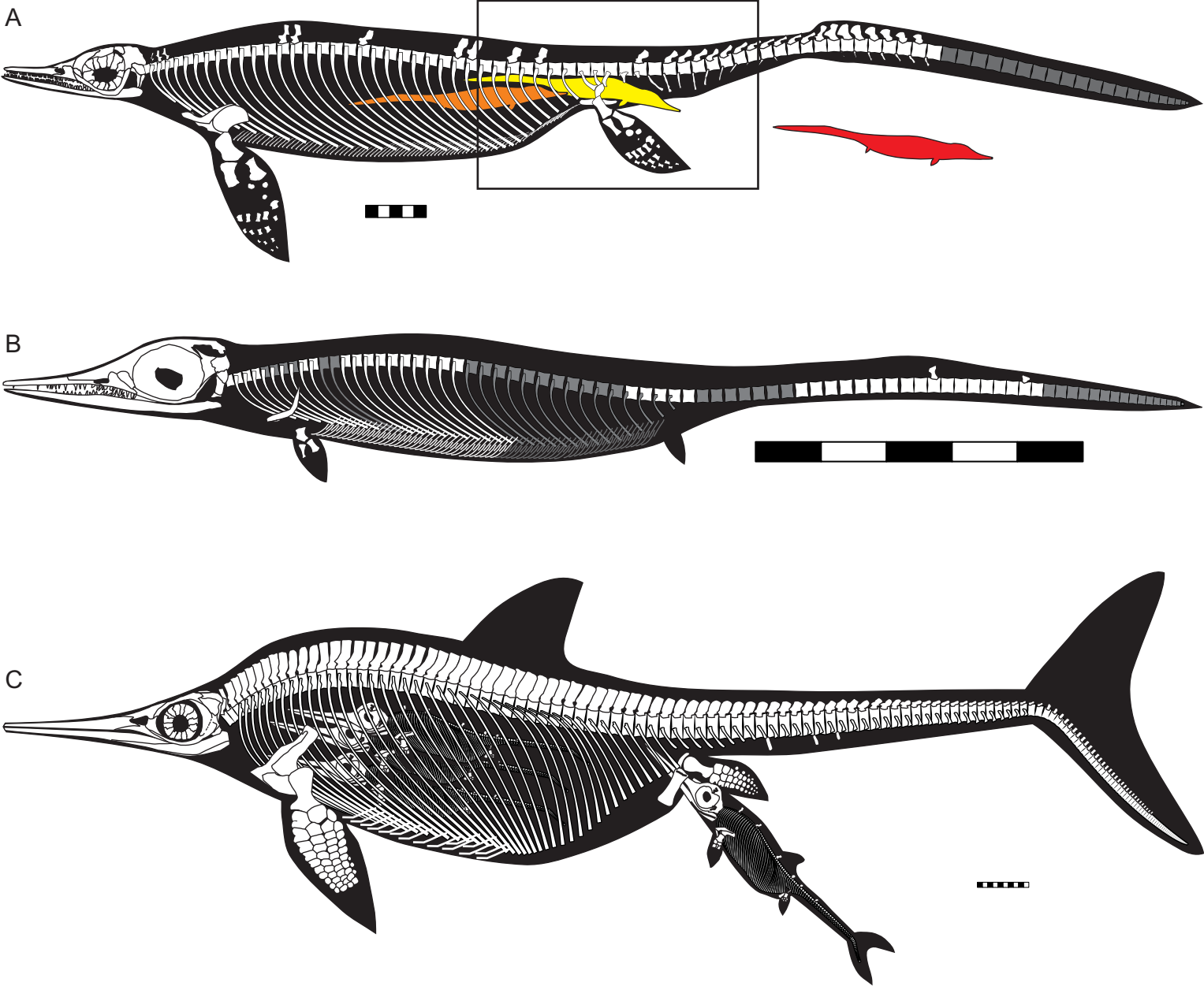

Supplement: Figure S3 — High resolution version of Fig. 4. (PDF) [file pone.0088640.s003.pdf]
